# Management of immunosuppression in lung transplant recipients and COVID-19 outcomes: an observational retrospective cohort-study

**DOI:** 10.1186/s12879-024-09269-1

**Published:** 2024-05-28

**Authors:** Hugo Bes-Berlandier, Benjamin Coiffard, Julien Bermudez, Nadine Demazes-dufeu, Bérengère Coltey, Céline Boschi, Philippe Colson, Sami Hraiech, Martine Reynaud-Gaubert, Nadim Cassir

**Affiliations:** 1 Department of Infectious Diseases, University Hospital Institute -Méditerranée Infection (IHU), Marseille, France; 2 Department of Respiratory Medicine and Lung Transplantation, Aix Marseille Univ, APHM, Hôpital Nord, Marseille, France; 3 Microbes, Evolution, Phylogeny and Infection (MEΦI), Hôpitaux de Marseille (AP-HM), Aix-Marseille Université, Institut de Recherche Pour le Développement IRD, Assistance Publique, Institut Hospitalo-Universitaire (IHU), Méditerranée Infection, 19-21 Boulevard Jean Moulin, Marseille, Cedex 05 13385 France; 4 Service de Médecine Intensive - Réanimation, AP-HM, Hôpital Nord, Marseille, France; 5 Faculté de médecine, Centre d’Etudes et de Recherches sur les Services de Santé et qualité de vie EA 3279, Aix-Marseille Université, Marseille, 13005 France

**Keywords:** Lung transplantation, COVID-19, SARS-CoV-2, Immunosuppression therapy, Risk factors

## Abstract

**Background:**

The aim of this study was to assess the impact of immunosuppression management on coronavirus disease 2019 (COVID-19) outcomes.

**Methods:**

We performed a single-center retrospective study in a cohort of 358 lung transplant recipients (LTx) over the period from March 2020 to April 2022. All included symptomatic patients had at least one positive SARS-CoV-2 rt-PCR. We used a composite primary outcome for COVID-19 including increased need for oxygen since the hospital admission, ICU transfer, and in-hospital mortality. We assessed by univariate and multivariate analyses the risk factors for poor outcomes.

**Results:**

Overall, we included 91 LTx who contracted COVID-19. The COVID-19 in-hospital mortality rate reached 4.4%. By hierarchical clustering, we found a strong and independent association between the composite poor outcome and the discontinuation of at least one immunosuppressive molecule among tacrolimus, cyclosporine, mycophenolate mofetil, and everolimus. Obesity (OR = 16, 95%CI (1.96; 167), *p* = 0.01) and chronic renal failure (OR = 4.6, 95%CI (1.4; 18), *p* = 0.01) were also independently associated with the composite poor outcome. Conversely, full vaccination was protective (OR = 0.23, 95%CI (0.046; 0.89), *p* = 0.047).

**Conclusion:**

The administration of immunosuppressive drugs such as tacrolimus, cyclocporine or everolimus can have a protective effect in LTx with COVID-19, probably related to their intrinsic antiviral capacity.

**Supplementary Information:**

The online version contains supplementary material available at 10.1186/s12879-024-09269-1.

## Introduction

Owing to their underlying comorbidities and immunosuppression, solid organ transplant recipients (SOTr) were feared to be at increased risk for worse outcomes from the coronavirus disease 2019 (COVID-19). However, contradictory data have been published on COVID-19 outcomes in lung transplant recipients (LTx) when compared to the general population [1, 2]. Indeed, Gatti et al. showed, in their recent meta-analysis, that there was no increased risk of mortality among SOTr, including 286 LTx, when adjusted for demographic, clinical features, and COVID-19 severity [3]. More recently, Trindade et al. found a mortality rate of 10% in 103 LTx who contracted COVID-19 between March 2020 and March 2022. When compared to the 6-month preceding the infection, the overall 6-month trajectory of lung function after infection did not change [4]. Besides, reports from the early phase of the pandemic recorded mortality rates exceeding 30% in New-York City from March to May, 2020 in LTx who contracted COVID-19 [5]. In 74 LTx from all transplant centers in the Netherlands who contracted COVID-19 between February 2020 and September 2021, the mortality rate was of 20% [6].

The achievement of a well-balanced immunosuppression is a complex challenge in LTx. Indeed, targeting a high level of immunosuppression helps to reduce the risk of rejection or chronic lung allograft rejection (CLAD) but also exposes to an increased risk of infection. According to international guidelines, the management of respiratory viral infections is based on a reduction or a discontinuation of immunosuppressive molecules in LTx presenting with moderate to severe disease [7]. While antimetabolite reduction with corticosteroid escalation has been a frequent strategy, there are no clear guidelines on how to handle immunosuppression in LTx infected by SARS-CoV-2 [8]. Recent studies suggested a protective effect of some immunosuppressive therapies due to their in vitro antiviral and anti-inflammatory properties, lessening the severity of the hyperinflammatory stage of COVID-19 [9]. In particular, tacrolimus, cyclosporine, and everolimus through inhibiting key cellular signaling pathways of SARS-CoV-2 replication and T-cell activation, could mitigate the cytokine storm syndrome associated with severe COVID-19 [10–12].

In this study, our main aim was to analyze the impact of immunosuppression management on the outcomes of COVID-19 in LTx from March 2020 to April 2022. We also described the epidemiology of COVID-19 in LTx in our Lung Transplant Center and analyzed factors associated with poor outcomes.

## Methods

### Design of the study

We conducted a single-center register-based retrospective observational study. The main aim was to analyze the impact of immunosuppression management on the outcomes of COVID-19 in LTx from March 2020 to April 2022. We also described the epidemiology of COVID-19 in LTx in our Lung Transplant Center and analyzed factors associated with poor outcomes.

### Patients

Symptomatic patients who contracted COVID-19 from March 2020 to April 2022 had to meet the following inclusion criteria: lung transplanted, aged ≥ 18 years old, follow-up in our lung transplant center (Marseille, France), presence of at least 1 positive SARS-CoV-2 rt-PCR test on nasopharyngeal swab. We used the Centers for Disease Control and Prevention (CDC) definitions for mild, moderate and severe COVID-19 [13]. Patients aged less than 18 years or having a positive SARS-CoV-2 PCR in the pre-transplant period, as well as those who had given an objection to the use of personal data for research purposes, were excluded from the analysis. LTx with COVID-19 were divided into 2 groups according to whether they met the criteria of a composite primary poor outcome including the increased need for oxygen (oxygen flow rate increased by at least 2 L per minute) since hospital admission, intensive care unit (ICU) transfer, and in-hospital mortality. We also recorded data on bacterial and fungal superinfection. The fulfillment of at least one factor was required to be classified in the poor outcome group.

The relief of the maintenance immunosuppression was defined by the discontinuation of at least one immunosuppressive molecule. The center’s usual immunosuppression protocol is included in the supplementary material. Each immunosuppressive molecule was either maintained at target doses, or suspended.

### Data collection

Data were collected using the computerized medical record. They were recorded at the time of COVID-19 diagnosis. Demographic characteristics collected included sex, age, medical history (obesity according to the World Health Organization (WHO) classification, arterial hypertension, cardiopathy, diabetes, chronic obstructive pulmonary disease (COPD), chronic renal failure (CRF) with glomerular filtration rate (GFR) < 60 ml/min/1.73 m2). Chronic lung allograft dysfunction (CLAD) in relation to bronchiolitis obliterans syndrome (BOS) was classified according to the CLAD BOS classification. Patients with CLAD BOS 1 and CLAD BOS 2 presented respectively a mild and moderate obstruction while patients with CLAD BOS 3 had a severe obstruction [14]. We have considered the onset of COVID-19 as the date of the first SARS-CoV-2 positive test. Regarding patients who had been infected several times by SARS-CoV-2 in the study period, we chose to consider only the more severe episode.

For patients without information about classification of SARS-CoV-2 into a lineage, we estimated the most probable variant based on the local SARS-CoV-2 epidemiologic survey. We thus defined several majoritarian variant periods as follows: Alpha variant (B.1.1.7) dominant period = March 2020 to June 2021, Delta variant (B.1.617.2) dominant period = July 2021 to December 2021, Omicron variant (B.1.1.529 or BA.1) dominant period = January 2022 to April 2022) (Figure S1) [15, 16]. If there was no dominant variant during the period of infection, the patient was considered in the category “no data”.

Patients were vaccinated with BNT162b2 vaccine or mRNA-1273 vaccine. The vaccination was considered complete if it included at least 3 doses [17].

According to the CDC guidelines, bacterial and fungal infections were considered as coinfection when they occurred simultaneously with COVID-19 and as superinfection when developed following a previous SARS-CoV-2 infection [18].

### Statistical analysis

Numerical variables were presented as median and interquartile range (IQR), while categorical variables as number and percentage. The statistical significance of differences was evaluated by chi-square or Fisher’s exact test for categorical variables and by Mann-Whitney U test for numerical variables. All tests were two-tailed. A p-value < 0.05 was considered significant. Analysis was performed using R software version 4.2.1 (R Foundation for Statistical Computing, Vienna, Austria) [19]. Items associated to outcomes at univariate analysis (P < 0.02) with clinical relevance were included in a multivariate logistic regression model to identify covariates independently associated with the composite poor outcome. The first multivariate analysis was based on a logistic regression model and included clinical variables (including gender, age, arterial hypertension, CRF, heart disease, obesity, diabetes, CLAD BOS and time between COVID-19 and transplantation) and vaccination status. The second model was a hierarchical ascendant classification on multiple correspondence analysis using the FactoMineR package (R software) [20]. The first step consisted in carrying out a multiple correspondence analysis which is a form of vector analysis that allows to describe a set of individuals using a range of qualitative variables. In this multiple correspondence analysis, we included the following variables: strain variant, the reason for transplantation and the management of the maintenance immunosuppression. Then, an unsupervised hierarchical classification was made to partition variables into clusters. The first cluster included all variables that were associated with the composite poor outcome and the second cluster includes all variables that were not associated with a poor outcome. Variables were assigned to each cluster according to their V statistic. Variables with a positive V statistic for the “poor outcome” cluster were assigned to this cluster while variables with a negative V-statistic for the “poor outcome” cluster were assigned to the “no poor outcome” cluster and were considered to be protective factors against the composite poor outcome. The V statistic number reflected the variable influence on the outcome, the more it increased, the more the association between the variable and the cluster was strong. In addition, we determined the ‘cla/mod’ for each variable, corresponding to the percentage of LTx having that modality in the cluster. Finally, the hierarchical ascendant classification led to the creation of a tree diagram (dendrogram) which illustrates the arrangement of clusters.

### Ethics

The study was registered in the Clinical Research and Innovation Department of Marseille Hospital (number: PADS20385) and was approved by the Ethics Committee of the Société de Pneumologie de Langue Française (CEPRO) (number CEPRO2023-036). It was not necessary to submit new requests to the French Data Protection Authority because the data were already collected in the context of healthcare.

## Results

**Fig. 1:**
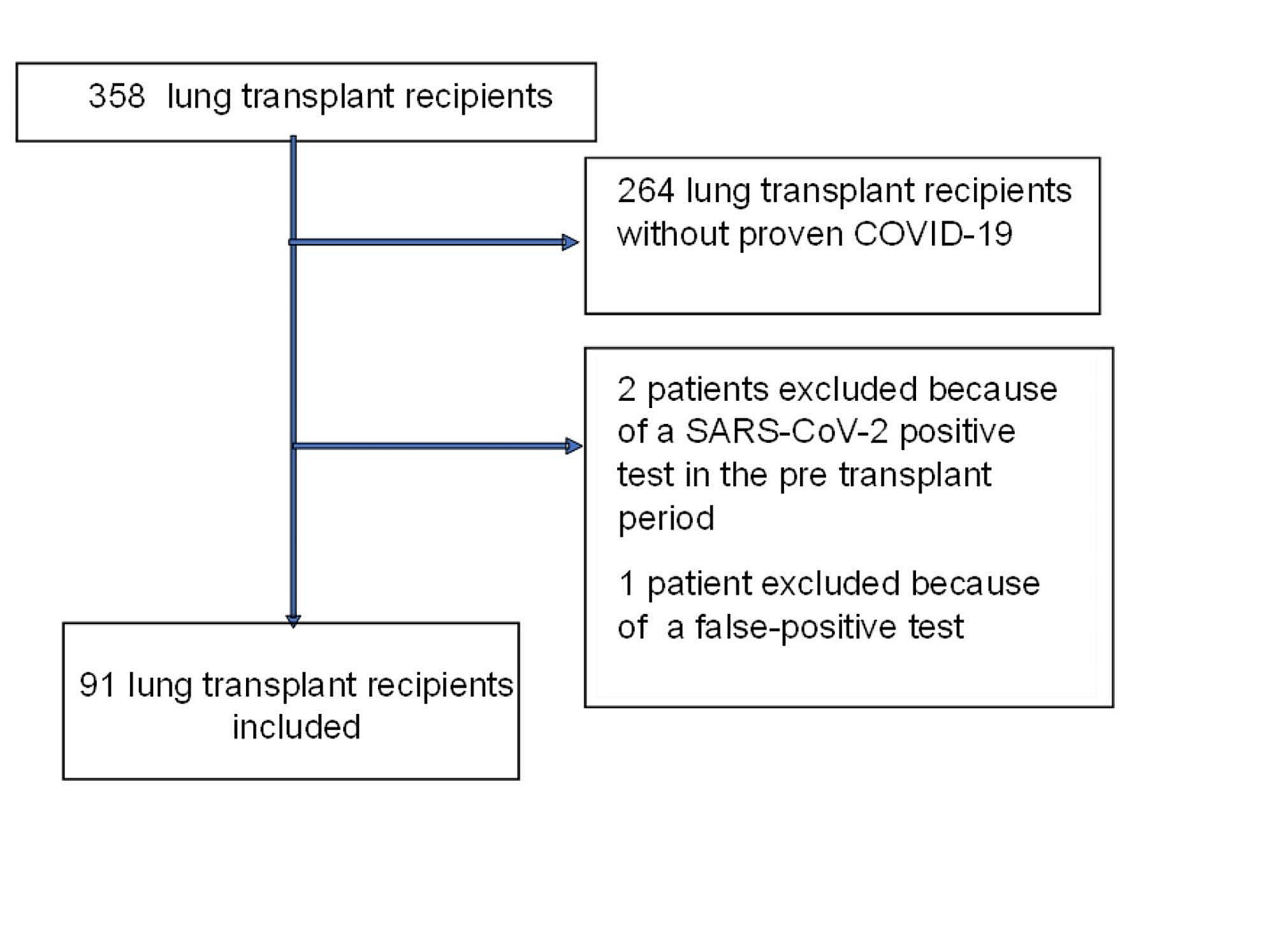
Flow chart. 91 lung transplant recipients with COVID-19 were included in this study

Overall, we included 91 (25%) LTx who contracted COVID-19 among the cohort of 358 LTx followed by our transplant team between March 2020 and April 2022 (Fig. 1). Forty-six (51%) patients were women and 45 (49%) were men. The median age was 51 years old and the median time from transplantation to the diagnosis of COVID-19 was 68 months. The reasons for transplantation were cystic fibrosis (33/91; 36%), COPD (24/91; 26%), pulmonary fibrosis (22/91; 24%), emphysema (7/91; 1%), pulmonary hypertension and bronchiectasis (5/91; 5%). Sixty-seven patients (74%) presented with mild disease, 6 (6%) had a moderate disease and 21 (20%) a severe disease. Thirty (33%) patients were hospitalized, including 10 (11) who had to be transferred to the ICU. The COVID-19 in-hospital mortality rate reached 4.4% (4/91) in our cohort (Table 1).

**Table 1:** Initial characteristics of lung transplanted recipients with COVID-19

Lung Transplant recipients’ characteristics	N = 91 (%)
**Sex**	
Male	45 (49)
Female	46 (51)
Median age [IQR]† in years	51 [35.5–61]
Time from transplantation to COVID-19 [IQR] † in months	68 [27–114]
**Reason for transplantation**	
Cystic fibrosis	33 (36)
COPD	31 (34)
Pulmonary fibrosis	22 (24)
Bronchiectasis or pulmonary hypertension	5 (5)
Alpha-1 antitrypsin deficiency	5 (5)
**COVID-19 symptoms**	
Mild	67 (74)
Moderate	6 (6)
Severe	21 (20)
**Hospitalization**	
Medicine department	20 (22)
ICU	10 (11)
Bacterial superinfection	11 (12)
Fungal superinfection	8 (9)

*Abbreviations* COPD = Chronic obstructive pulmonary disease, ND = no data, ICU = Intensive care unit

† IQR: interquartile range

Among the 91 LTx who contracted COVID-19, 18 (20%) had at least one criteria of the composite primary poor outcome. Among patients in the poor outcome group, 18 required an increased need for oxygen (oxygen flow rate increased by at least 2 L per minute) since hospital admission, 9 were transferred to the ICU, and 4 died. Bacterial superinfections were documented in 11 (12%) patients. In addition, 8 (9%) patients presented a fungal superinfection of which 7 (8%) with filamentous fungi (Table 1). Fungal infections caused by *Aspergillus* sp. included four invasive pulmonary infections and two tracheobronchitis.

By univariate analysis, no significant differences were observed between gender or age and the poor composite outcome. Cystic fibrosis (CF) as a reason for transplantation, full anti-SARS-CoV-2 vaccination, being infected by the Omicron variant and immunosuppression maintenance were significantly protective against the composite poor outcome. Conversely, being infected by the Delta or pangolin B.1.177 variant, acute kidney injury (AKI), everolimus use in the baseline immunosuppressive treatment and the relief of at least one immunosuppressive treatment were significantly associated with the composite poor outcome. Moreover, we did not find any association between the use of Casirivimab-imdevimab and a poor outcome (Table 2).

**Table 2:** Risk factors for poor primary composite outcome by univariate analysis

	No poor outcome *N* = 73 (%)	Poor outcome *N* = 18 (%)	*P* value
**Sex**			
Male	34 (46)	11 (61)	
Female	39 (54)	7 (39)	0.27
Age [IQR]† in year	51 [34–60]	54.5 [41.5-62.25]	0.22
Time from Transplantation to COVID-19 [IQR]† in months	77 [29–115]	42 [12-64.25]	0.038
Transplanted within 12 months prior to COVID-19	10 (14)	5 (28)	0.045
**Reason for lung transplantation**			
Cystic fibrosis	31 (43)	2 (11)	0.01
COPD	23 (31)	8 (44)	0.41
Pulmonary Fibrosis	16 (22)	6 (33)	0.36
Alpha-1 antitrypsin deficiency	3 (4)	2 (11)	0.26
**Comorbidities**			
Arterial hypertension	42 (58)	14 (78)	0.17
Diabetes	38 (52)	8 (44)	0.6
CRF ‡	21 (29)	9 (50)	0.086
Obesity ^a^	3 (4)	3 (17)	0.1
Cardiopathy ^b^	6 (8)	2 (11)	0.65
CLAD BOS 1 or 2 §	16 (21)	1 (6)	0.17
CLAD BOS 3 §	3 (4)	2 (11)	1
Recent treatment for chronic humoral rejection	2 (3)	0	1
Recent treatment for acute rejection	2 (3)	1 (6)	0.49
**Baseline immunosuppression**			
Tacrolimus	72 (99)	18 (100)	0.35
Cyclosporine	1 (1)	0	0.2
MMF or azathioprin	66 (82)	12 (67)	0.01
Everolimus	6 (8)	5 (28)	0.04
Oral corticosteroids	21 (29)	8 (44)	0.2
**Number of immunosuppressive molecules**			
1	4 (5)	1 (6)	0.38
2	45 (62)	11 (61)	
3	22 (30)	4 (22)	
4	2 (3)	2 (11)	
Discontinuation of at least one immunosuppressive molecule	2 (3)	13 (72)	< 0.001
No AKI	68 (93)	5 (28)	< 0.001
AKI without dialysis	5 (7)	11 (72)	
AKI requiring dialysis	0	2 (18)	
**Variant**			
French original B.1.1	3 (4)	1 (6)	1
Pangolin B.1.177	0 (10)	2 (11)	0.03
Pangolin B.1.160	8 (11)	2 (11)	1
Alpha	5 (7)	0	0.58
Delta	10 (14)	9 (50)	0.0007
Omicron	43 (59)	4 (22)	0.007
Omicron BA.1	22 (30)	3 (75)	0.34
Omicron BA.2	21 (29)	1 (25)	
ND	4 (4)	0	0.58
**Vaccine**			
Unvaccinated	18 (25)	8 (47)	0.096
Incompletely vaccinated	7 (10)	3 (16)	0.43
Fully vaccinated	48 (66)	7 (39)	0.037
Tixagevimab-cilgavimab prophylaxis	11 (15)	3 (16)	1
Casirivimab-imdevimab prophylaxis	12 (16)	5 (28)	0.34
Bacterial superinfection	1 (1)	10 (56)	< 0.001
*Pseudomonas aeruginosa*	1(100)	6 (60)	
*Escherichia coli*	0	1 (10)	
*Klebsiella pneumoniae*	0	1 (10)	
*Enterobacter aerogenes*	0	1 (10)	
*Staphyloccocus aureus*	0	2 (20)	
*Staphyloccocus haemolyticus*	0	1 (10)	
*Stenotrophomonas maltophila*	0	1 (10)	
*Enterococcus faecalis*	0	1 (10)	
Fungal superinfection	0	8 (44)	< 0.001
*Candida* sp.		1 (12.5)	
*Aspergillus* sp.		6 (75)	
*Mucor* sp.		1 (12.5)	
Intrahospital mortality	0	4 (22)	< 0.001

*Abbreviation* ND = no data, MMF = Mycophenolate Mofetil, COPD = Chronic Obstructive Pulmonary Disease, AKI = Acute Kidney Injury

† IQR: interquartile range

‡ CRF = chronic renal failure, defined by a glomerular filtration rate of less than 60 ml/min/1.73m2

^a^ Obesity was defined by a body mass index superior or equal to 30

^b^ Cardiopathy included ischemic heart disease and dilated cardiomyopathy

^c^ All patients were treated at preventive dose except for two patients in poor outcome group and one in good outcome group

§CLAD BOS = Chronic lung allograft dysfunction in relation to bronchiolitis obliterans syndrome. Patients with CLAD BOS 1 and CLAD BOS 2 presented respectively a mild and moderate obstruction while patients with CLAD BOS 3 had a severe obstruction

In the multivariate analysis including clinical variables and vaccination, obesity (OR = 16, 95%CI (1.96; 167), *p* = 0.01) and chronic renal failure (OR = 4.6, 95%CI (1.4; 18), *p* = 0.01) were independently associated with a composite poor outcome. Conversely, full vaccination was protective against a composite poor outcome (OR = 0.23, 95%CI (0.046; 0.89), *p* = 0.047) (Table 3).

**Table 3:** Multivariate analysis of risk factors for poor primary composite outcome after by Logistic regression

Variable	OR (95% CI)	*P* value
Full vaccination	0.23 (0.046–0.89)	0.047
Obesity	16 (1.96–167)	0.01
CRF	4.6 (1.4–18)	0.01

OR: Odds ratio, CI = confidence interval, CRF = chronic renal failure

The hierarchical ascendant classification of individuals yielded two clusters. The “poor outcome” cluster included by decreased order of association: tacrolimus or ciclosporin discontinuation, AKI, MMF or azathioprine disontinuation, everolimus discontinuation, and being infected by the Delta SARS-CoV-2 (Table 4; Figure S2). We could not provide definitive data on the timing of the modification in relation to the initial time of infection. The “no poor outcome” cluster included by decreased order of association: tacrolimus or ciclosporin high dose continuation, being infected by the Omicron SARS-CoV-2 variant, and CF as a reason for transplantation (Table 4).

**Table 4:** Factors associated with the primary composite outcome after ascending hierarchical clustering

	Cla/mod (%)	V test
Tacrolimus or cyclosporine continuation	96	-7
Omicron variant period	94	-2.7
Cystic fibrosis	97	-2.6
Delta variant	58	3
Everolimus discontinuation	100	4
MMF/azathioprine discontinuation	78	4.2
Acute kidney injury	72	6.2
Tacrolimus or cyclosporine discontinuation	100	7.1

## Discussion

We showed in this study that the relief of the maintenance immunosuppressive regimen was associated with a poor composite outcome in LTx with COVID-19. By univariate analysis, we showed an association between the everolimus use in the basal immunosuppressive maintenance regimen and the composite poor outcome. However, this result is biased by confounding factors. Indeed, everolimus was more frequently prescribed in LTx who experienced poor tolerance to calcineurin inhibitors (renal failure) and with more comorbidities. Notably, a large international survey who analyzed the management of the immunosuppressive treatment in several LTx centers showed that most clinicians had continued calcineurin inhibitors and mTOR inhibitors in their patients diagnosed with COVID-19 [21]. By hierarchical clustering, we found a strong and independent association between the relief of tacrolimus, MMF or azathioprine, and everolimus corresponding to a reduction in dose or suspension and the composite poor outcome. These results are consistent with studies in other SOT populations which highlighted a decreased risk of severe disease and death in recipients treated with everolimus as part of the maintenance immunosuppressive regimen [22, 23]. In the same way, Belli et al. described an association between the continuation of tacrolimus and better survival in liver transplant recipients [24]. This beneficial effect of immunosuppressive treatment was also observed by Desmazes-Dufeu et al. who reported the case of COVID-19 in a cohabiting couple of LTx and showed a better clinical course in the patient recently transplanted (18 months ago *versus* 13 years ago) which was also characterized by a higher level of immunosuppression [25]. Immunosuppressive molecules used as maintenance immunosuppressive therapy in LTx have been studied for their antiviral and anti-inflammatory properties. For instance, mTOR inhibitors were shown to inhibit viral translation by blocking the mTOR pathway which takes action in key cellular signaling pathways for SARS-CoV-2 [23, 26]. Moreover, the mTOR inhibitors could mitigate the immune response hyper-activation associated with the inflammatory phase of COVID-19 [27]. Tacrolimus could also reduce the production of many proinflammatory cytokines by suppressing the early phase of T-cell activation and inhibiting the growth of some coronavirus by targeting the immunophilin FK506 binding proteins [12, 28, 29]. Besides, ciclosporin could be in a similar way helpful on COVID-19 courses through its in vitro activity against SARS-CoV-2 and its immunomodulatory function by reducing IL2 production [11, 30, 31]. In previous cohorts of COVID-19 in SOTr, improved survival was seen in those who continued calcineurin inhibitors during infection compared with those who discontinued calcineurin inhibitors [32, 33]. The protective role of MMF and/or azathioprine is less comprehensive and needs to be confirmed in further work. Indeed, the use of MMF has been associated with poor outcomes after COVID-19 in renal transplant recipients [34]. However, in a cohort of patients with neuromyelitis optica spectrum disorder, mostly treated with MMF or azathioprine, the duration of COVID-19 symptoms was shorter and the clinical symptoms were less severe than those reported in the general population [35]. The authors hypothesised that this could be partly explained by inhibition of the JAKs pathway, TNF, IL-1 and granulocyte-macrophage colony-stimulating factor, all of which are involved in the COVID-19-related cytokine storm syndrome.

In our cohort of 91 LTx diagnosed with COVID-19, the mortality rate was low (4%) and similar to that of LTx without COVID-19 (*N* = 267) during the study period from March 2020 to April 2022. These data are consistent with previous studies suggesting that SOTr are not at increased risk of COVID-19 poor outcome although contradictory results have been published [36]. Several factors could explain the discordance in mortality and reported long-term respiratory function outcomes in LTx cohorts. First, the COVID-19 pandemic has been characterized by successive waves caused by different SARS-CoV-2 variants [37] associated with different short and long-term outcomes [38]. In our cohort, by multivariate analysis, being infected by the Omicron SARS-CoV-2 variant was significantly associated with a decreased risk of poor outcome while being infected by the Delta and Pangolin B.1.177 SARS-CoV-2 variants was significantly associated with an increased risk of poor outcome. These observations are consistent with other works that reported lower mortality rates in SOTr affected by the Omicron variant and in congruence with what has been observed in the general population [39]. Second, reports from the early phase of the COVID-19 pandemic inevitably included a higher percentage of patients without or with partial SARS-CoV-2 immunization protection. Third, treatment strategies provided for COVID-19 have also strongly evolved during our study period. Finally, comorbidities also influenced the prognosis as shown by the association in multivariate analysis between obesity and poor outcome which has been as well described by Messika et al. [40]. It is worth noting that Heldman et al. highlighted a trend toward increased mortality among LTx with obesity [22]. Nevertheless, the risk of a worse outcome for patients with chronic kidney disease found in our study is in contradiction with works published by Kamp et al. and Heldman et al. who concluded that there was no association between chronic kidney disease and mortality in LTx [22, 41].

We acknowledge that our study presents several limitations. First, the retrospective design may have caused bias, especially a risk of confounding by indication illustrated by the choice of clinician to relief the immunosuppressive treatment in severe COVID-19. We could not provide definitive data on the timing of immune modification in relation to the initial time of infection. The reduction in immunosuppression may itself be a marker of more severe COVID-19 and induce the physician to reduce its levels at the time of diagnosis. However, our composite primary outcome included endpoints that were all evaluated after the maintenance immunosuppressive treatment modification. Second, the long recruitment period led to significant heterogeneity in our population, reason why we did not integrate in our analyses the treatments that were provided for COVID-19. Finally, our results could not be extrapolated to other respiratory viruses and further studies are needed to better understand the impact of immunosuppression management on infections caused by each respiratory virus.

## Conclusions

The administration of immunosuppressive drugs such as tacrolimus, cyclocporine or everolimus may have a protective effect in LTx with COVID-19, probably related to their intrinsic antiviral capacity. Further prospective and large-scale studies would be needed to confirm these results before providing comprehensive guidelines on immunosuppression management in LTx with COVID-19.

### Electronic supplementary material

Below is the link to the electronic supplementary material.

Supplementary Material 1

## Data Availability

The data that support the findings of this study are available from the corresponding author upon reasonable request.
