# Supplementary material for: Management of immunosuppression in lung transplant recipients and COVID-19 outcomes: an observational retrospective cohort-study
Source: BMC Infect Dis. 2024 May 28;24:536. doi: 10.1186/s12879-024-09269-1 (PMC11134755; doi:10.1186/s12879-024-09269-1)

**Supplemental material**

**Center’s usual immunodeppression protocol:**

Induction therapy consisted of basiliximab for all recipients, combined with high-dose methylprednisolone (10 mg/kg/day). A standard triple maintenance regimen included tacrolimus (maintained at trough blood levels between 12 and 14 ng/ml for the first 12 months and then approximately 8–12 ng/ml), mycophenolate mofetil (2g/day and then adjusted to trough blood levels between 1. 5 to 4 µg/mL) and steroids (1 mg/kg/day for the first 10 days, tapered to 0.5 mg/kg/day during the first month, and further tapered to 5–10 mg/day for approximately one year). In cases of calcineurin inhibitor-induced chronic renal failure, everolimus (maintained at trough blood levels between 3 and 8 ng/mL) is introduced after complete healing of the bronchial anastomoses is assured. This introduction allows tacrolimus to be reduced to a trough blood level of approximately 4–5 ng/mL.

**Specific treatment in 91 lung transplanted recipients with COVID-19, based on evolving guidelines and molecules availability :**

| Oral or intravenous dexamethasone (6mg/day)  Intravenous methylprednisolone (1mg/kg/day) | 16 (18%)  3 (3%) |
| --- | --- |
| Tocilizumab  Convalescent Plasma  Sotrovimab  Casirivimab-Imdevimab  Tixagevimab/Cilgavimab | 2 (2%)  1 (1%)  9 (10%)  20 (22%)  28 (31%) |

**Figure S1 :** Weekly incidence of each SARS-CoV-2 mutants and variants extrapolated to the total number of cases, based on their proportions of genotyped cases, among patients SARS-CoV-2-diagnosed at IHU Méditerranée Infection institute.


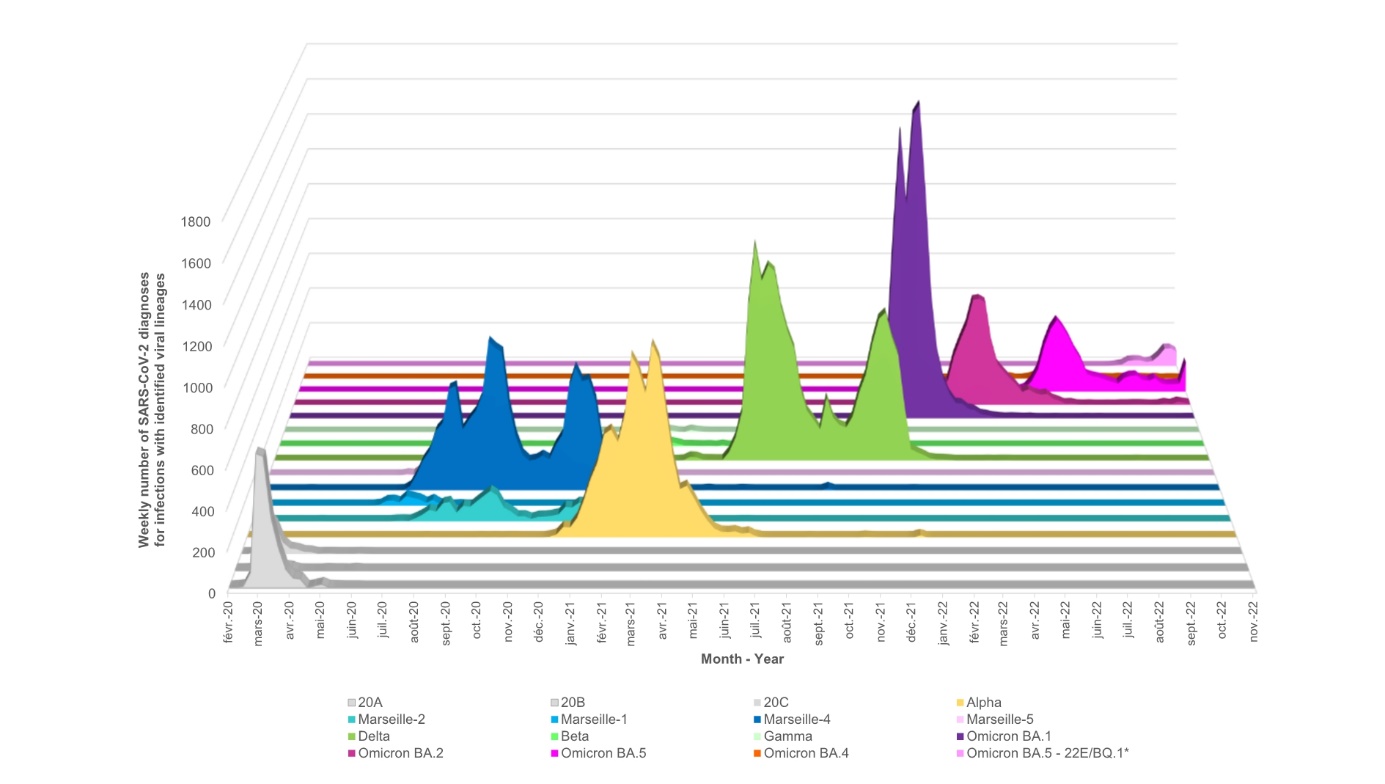


**Figure S2: Hierarchical ascending classification of patient presented in a dendogram cluster analysis.** Lung transplant recipients are plotted on the abscissa axis and variables are plotted on the ordinate axis. The black and red rectangles define the two clusters of patients. The first cluster (**red rectangle**) gathered variables associated with a COVID-19 poor outcome: being infected by Delta variant and the relief of at least one immunosuppressive molecule among tacrolimus, ciclosporin, everolimus, MMF, or azathioprine. The second cluster (**black rectangle)** was composed by all variables protective against a COVID-19 poor outcome: being affected by cystic fibrosis, tacrolimus or ciclosporin continuation, the absence of acute kidney disease and being infected by the Omicron variant. The graph in the top right corner represents the loss of inertia according to the different dimensions.


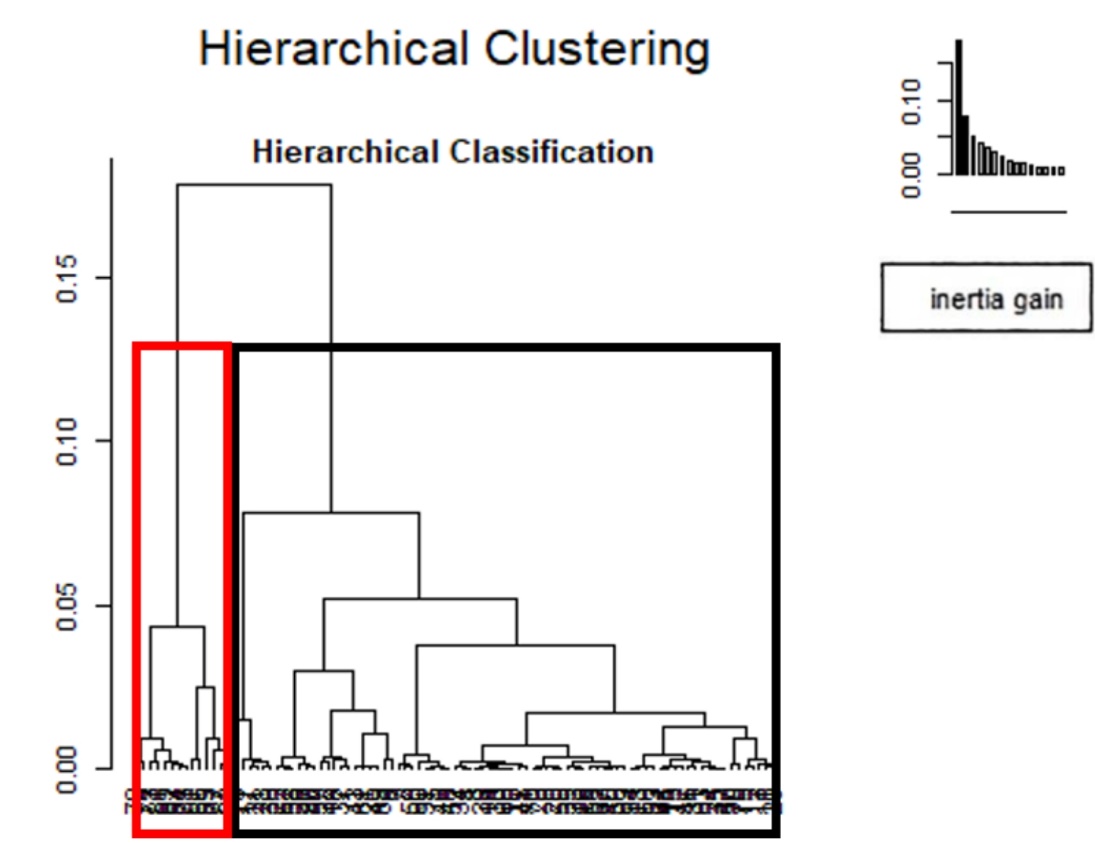

Supplement: Supplementary file 1 — Supplementary Material 1 [file 12879_2024_9269_MOESM1_ESM.docx]
